# Modeling Robustness Tradeoffs in Yeast Cell Polarization Induced by Spatial Gradients

**DOI:** 10.1371/journal.pone.0003103

**Published:** 2008-09-01

**Authors:** Ching-Shan Chou, Qing Nie, Tau-Mu Yi

**Affiliations:** 1 Department of Mathematics, Center for Mathematical and Computational Biology, Center for Complex Biological Systems, University of California Irvine, Irvine, California, United States of America; 2 Department of Developmental and Cell Biology, Center for Complex Biological Systems, University of California Irvine, Irvine, California, United States of America; Duke University Medical Centre, United States of America

## Abstract

Cells localize (polarize) internal components to specific locations in response to external signals such as spatial gradients. For example, yeast cells form a mating projection toward the source of mating pheromone. There are specific challenges associated with cell polarization including amplification of shallow external gradients of ligand to produce steep internal gradients of protein components (e.g. localized distribution), response over a broad range of ligand concentrations, and tracking of moving signal sources. In this work, we investigated the tradeoffs among these performance objectives using a generic model that captures the basic spatial dynamics of polarization in yeast cells, which are small. We varied the positive feedback, cooperativity, and diffusion coefficients in the model to explore the nature of this tradeoff. Increasing the positive feedback gain resulted in better amplification, but also produced multiple steady-states and hysteresis that prevented the tracking of directional changes of the gradient. Feedforward/feedback coincidence detection in the positive feedback loop and multi-stage amplification both improved tracking with only a modest loss of amplification. Surprisingly, we found that introducing lateral surface diffusion increased the robustness of polarization and collapsed the multiple steady-states to a single steady-state at the cost of a reduction in polarization. Finally, in a more mechanistic model of yeast cell polarization, a surface diffusion coefficient between 0.01 and 0.001 µm^2^/s produced the best polarization performance, and this range is close to the measured value. The model also showed good gradient-sensitivity and dynamic range. This research is significant because it provides an in-depth analysis of the performance tradeoffs that confront biological systems that sense and respond to chemical spatial gradients, proposes strategies for balancing this tradeoff, highlights the critical role of lateral diffusion of proteins in the membrane on the robustness of polarization, and furnishes a framework for future spatial models of yeast cell polarization.

## Introduction

Breaking symmetry is a fundamental process in biology [1]. Components that were previously uniformly distributed become asymmetrically localized. This anisotropy or polarization creates complexity of form and function. The challenge is polarizing in the right place at the right time to the proper extent under uncertain and changing conditions (i.e. robust polarization).

Cells localize components to specific locations leading to morphological changes in response to internal and external cues. For example, haploid cells of the yeast *Saccharomyces cerevisiae* typically form a new bud at the site of the previous bud (internal cue). In addition, haploid yeast cells can sense an external gradient of mating pheromone and form a mating projection (shmoo) toward the source. In both cases, a large number of signaling, structural, and transport proteins localize at the site of the morphological change [2], [3].

There has been extensive mathematical modeling of cell polarization as a special case of pattern formation in living systems. Turing originally proposed that complex spatial patterns could arise from simple reaction-diffusion systems [4]. Meinhardt popularized the modeling of biological pattern formation using generic reaction-diffusion models. In particular, he introduced the idea that polar structures could arise from local autocatalysis (i.e. positive feedback) balanced by global inhibition [5]. Subsequently, researchers constructed more detailed models that incorporated information about specific molecular species and reactions in cells undergoing chemotaxis. One class of models used a local excitation, global inhibition (LEGI) mechanism [6], [7].

**Figure 1:**
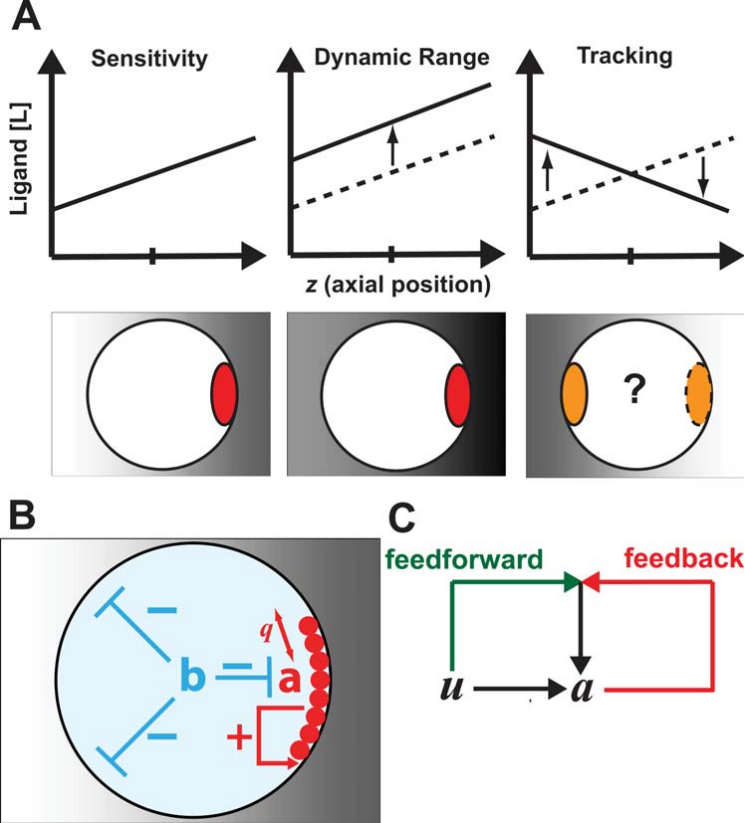
Schematic descriptions of performance objectives and model of polarization. (A) Performance objectives of sensing and responding to a gradient. The graphs depict the concentration of chemical ligand along the axial length of the cell. Below each graph is a picture of a cell in a chemical gradient (background shading) with the polarized component in red. The chemical gradient may be shallow (sensitivity), the average concentration may be low or high (dynamic range), and the direction of the gradient may be changing (tracking). In each case, the external gradient must be amplified to create a polarized distribution of some internal component. (B) In the model, the polarized species *a* (red) becomes localized to the front of the cell through cooperative interactions (*q* is the Hill cooperativity parameter) in response to the input and through positive feedback (+). There is global negative feedback (integral control) mediated by the species *b* (blue). (C) In feedforward/feedback coincidence detection, the positive feedback amplification of *a* depends on a feedforward component originating from the input *u* (green) and a feedback component originating from *a* (red).

Sensing and responding to a chemical gradient present many challenges including sensitivity, dynamic range, tracking, and noise (Fig. 1A). The gradient may be shallow and the concentration difference between front and back small (sensitivity). The average concentration of the chemical ligand may be much higher or lower than the dissociation constant (*K_d_*) of the ligand receptor (dynamic range). The source of the chemical signal may be moving (tracking). There may be noise in the gradient, and so forth. It is an open question how well these different performance objectives can be achieved simultaneously. In the literature, the focus has been on understanding how a shallow external gradient can be amplified to create a steep internal gradient of cellular components. High amplification can result in an all-or-none localization of the internal component to a narrow region. However, the tracking of a moving signal source has also been acknowledged to be important. Devreotes and colleagues [8] made the distinction between directional sensing (low amplification, good tracking) and polarization (high amplification, poor tracking). Meinhardt first highlighted the potential tradeoff between amplification and tracking [9].

This field possesses an extensive literature, and Dawes et al. [10] reviewed a number of previous models of eukaryotic gradient-sensing and cell polarization. Included were the models of Meinhardt [9], Narang [11], Levchenko-Iglesias [12], Postma-Van Haastert [13], Maly et al. [14], Haugh and colleagues [15], Gamba et al. [16], and Skupsky et al. [17]. Many of the models contained some type of positive feedback structure, as well as nonlinearities capable of generating ultrasensitivity to the input. The models ranged from generic models (e.g. [9], [11], [13] ) to more mechanistic models (e.g. [10], [14], [17]).

Dawes et al. categorized the models according to gradient-sensing, amplification, polarization, tracking of directional change, persistence when the stimulus is removed (i.e. multi-stability), etc. Among the 23 papers containing models mentioned in the article, only four [9], [10], [17], [18] simultaneously considered the issues of amplification, tracking, and multi-stability. Of these 4, the paper by Skupsky et al. [17] was most related to the work described here. Those authors defined 4 modes of gradient-sensing that depended on the strength of the positive feedback and the extent of translocation of signaling molecules from the cytoplasm to the membrane. These modes varied in the degree of amplification (polarization), presence of multiple steady-states, response to a rotating gradient, etc. However, a detailed characterization of the modes was hampered by the complexity of the mechanistic model. We have presented a more mathematical treatment using generic models motivated by yeast (small) cell gradient-sensing and polarization. These simple models motivated more complex mechanistic models later in our paper.

Here we investigated in a systematic fashion the tradeoff between amplification and tracking during gradient-sensing. We demonstrated the nature of these tradeoffs using a simple model and well-defined measures of performance. In particular, we focused on the roles of cooperativity and positive feedback on amplification and their effects on tracking. Although the tradeoff could not be eliminated, it could be fine-tuned through modifications to the model to ensure balanced performance in specific regimes of external conditions. In addition, we demonstrated that moderate lateral surface diffusion in the membrane increased the robustness of polarization. Finally, we used these findings to update our previous model of yeast spatial sensing of mating pheromone, and simulate polarization for a range of surface diffusion coefficients.

## Results

### Generic Model and Measures of Polarization and Amplification

Previously we constructed a model of yeast cell polarization that explicitly represented spatial dynamics [19]. In that model we explored the tradeoff between amplification of a shallow external gradient into a steeper internal gradient of intracellular components and tracking a gradient whose direction is changing. Both objectives were hard to achieve simultaneously. The complexity of the model, however, prevented a thorough analysis of the tradeoff. Here, we constructed a simpler, generic model that captured the essence of the larger model.
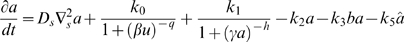
(1.1)

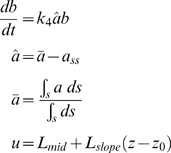
(1.2)


The default value for most of the parameters was 1: *k*_0_ = *k*_1_ = *k*_2_ = *k*_3_ = *k*_4_ = *k*_5_ = 1 s^−1^; *β* = *γ* = 1; *a_ss_* = 1. This default case assumes that all of the dynamics in the system are on the same time-scale. In the investigations below, we typically varied the values of *k*_0_, *k*_1_, *q*, *h* and *D_s_*. We also explored varying the other parameters (data not shown) but found that they did not impact the steady-state behavior as significantly. The input *u* and the variables *a* and *b* were chosen to be unitless.

In this model (Model 1), the variable *a* represents the concentration of the species undergoing polarization and whose spatial dynamics are of interest (Fig. 1B). The second variable *b* represents the concentration of a negative regulator involved in a negative feedback loop that regulates *a* and behaves like a global inhibitor; it is uniformly distributed throughout the cell. The input *u* is a linear chemical gradient. The species represented by *a* is assumed to be bound to the membrane and the term 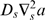 describes its lateral surface diffusion in the membrane with diffusion coefficient *D_s_*. The second term (*k*_0_/1+(*βu*)^−*q*^) in Eq. (1.1) represents the cooperative production of *a* which depends on the input *u*; the form of the term is a Hill expression possessing a Hill cooperativity parameter *q* and a Hill half-maximal constant 1/*β*. The third term is a positive feedback term in which *a* stimulates its own production. This autocatalytic reaction is also a cooperative reaction possessing a Hill cooperativity parameter *h* and a Hill half-maximal constant 1/*γ*.

Degradation is described by a first-order decay term (*k*_2_ *a*). Regulation is achieved through two negative feedback terms representing proportional feedback (*k*_5_ *â*) and integral feedback (*k*_3_ *ba*) [20]. The variable *b* is involved in the integral feedback control loop with the second differential equation ensuring that the average steady-state levels of *a* (*a̅*) will tend to the fixed value *a_ss_*. The variable *â* represents the difference between *a̅* and *a_ss_*. Because the integral feedback term *k*_3_ *ba* cannot be negative, the steady-state concentration of *a* will drop below *a_ss_* for low input values. Note that we have assumed that there is fast mixing of the negative regulator represented by *b* in the cell interior; this assumption is likely to be valid for smaller cells. In addition, we point out that the production of the negative regulator *b* is autocatalytic, which prevents *b* from becoming negative. Finally, modifying the form of Eq. (1.2) by adding a constant basal synthesis rate (*k_6_*) for *b* breaks the integral control, but did not significantly alter the steady-state behavior of the model.

Geometrically, we modeled the cell as a sphere with radius 1 µm. We applied a linear spatial gradient described by the concentration of ligand at the center of the cell, *L_mid_*, and the gradient slope *L_slope_* (which was relative to *L_mid_*); *z* is the axial coordinate along the length of the cell in the direction of the gradient and *z*_0_ is the position of the center of the cell. The response of the cell was measured by the spatial dynamics of *a*, and in particular, the polarized distribution of *a*. We represented these dynamics in one-dimension (1D) along the axial length because a sphere is rotationally symmetric around its axis (axisymmetric).

Biologically, we interpret this model as a signal transduction cascade in which the cooperative assembly of multi-protein signaling complexes can give rise to the cooperative input term. Positive feedback is found in many of these signaling systems. For example, in the yeast mating response the combined actions of the proteins Bem1p, Cdc42p, and Cdc24p create a positive feedback loop [21]. Negative feedback loops are also ubiquitous in signaling pathways and can act upstream at the level of receptor down-regulation to the more downstream transcriptional activation of negative regulators. Thus, we view the model as a simplification of more sophisticated models of cell polarity and chemotaxis from other authors such as the LEGI models previously mentioned [6]. It is important to note that for simplicity we chose a generic model formalism that does not obey mass-action. For example, the synthesis terms show no dependence on the “substrate” of *a*, implying that the level of substrate is constant. However, the fundamental spatial dynamics of the generic model are reproduced in mass-action models such as the model of yeast pheromone-induced polarization described later.

We investigated several measures of polarization. First was the value of *a* at the front of the cell, *a_f_*, where the concentration of ligand is highest. The second was an approximation of the relative slope of *a*:
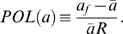



The average concentration of *a* is *a̅*, and the radius of the cell is *R*. The third measure termed the polarization factor (*PF*) describes the “width” of the global distribution of the polarized component:
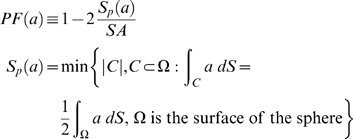

*S_p_*(*a*) is the surface area at the front of the cell that encompasses 50% of the polarized component *a* and *SA* is the total surface area of the cell. An unpolarized cell would have a *PF* of 0 and an infinitely polarized cell would have a *PF* of 1. We concluded that in most cases, all three measures conveyed the same information (data not shown), and we have typically plotted *a_f_* for convenience and consistency.

Amplification refers to the conversion of the external gradient signal into the polarization of the internal component. We defined the amplification factor (*AF*) as the ratio of polarization of *a* to the relative slope (i.e. polarization) of the external gradient of ligand *L* (*POL*(*L*)). A large *AF* indicates that the cell can amplify a shallow spatial gradient to produce significant internal polarization:
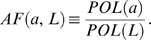



### Amplification is Produced by Cooperativity or Positive Feedback

**Figure 2:**
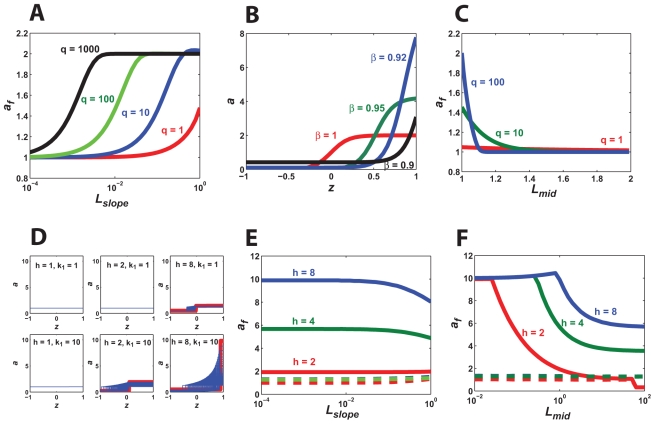
Cooperativity and positive feedback produced polarization and amplification. (A) Cooperativity alone (*k*_1_ = 0) resulted in amplification of shallow gradients for larger values of *q*. Polarization was measured by *a_f_*, the value of *a* at the front of the cell. We plotted polarization as the slope of the external gradient, *L_slope_*, was varied (*L_mid_* = 1). (B) With cooperativity alone, polarization increased by fine-tuning the Hill constant *β*. The variable *a* was plotted as a function of position along the axial length *z*. *L_mid_* = 1, *L_slope_* = 0.1 µm^−1^, *k*_0_ = 10 s^−1^, *q* = 100. (C) Cooperativity alone showed limited dynamic range. We varied *L_mid_* over a narrow range from 1 to 2 (*L_slope_* = (0.1×*L_mid_*) µm^−1^) and determined *a_f_* for different values of *q*. (D) Increasing the positive feedback gain (*k*_1_>0, *h*≥1) enhanced polarization and produced multiple steady-states. We plotted the envelope of possible steady-state solutions. For cases with multiple solutions, the red trace represents the maximum polarization solution in the envelope. (E) Infinite amplification of shallow gradients with positive feedback. For *k*_1_ = 10 s^−1^, we plotted *a_f_* for three values of *h* as we varied the gradient slope (*L_mid_* = 1). The dashed lines represent the minimum polarization in the solution envelope for each value of *h*. (F) Positive feedback produced broad dynamic range. We plotted *a_f_* as a function of *L_mid_* (*L_slope_* = 0.01 µm^−1^) for three values of *h* (*k*_1_ = 10 s^−1^). Dashed lines represent minimum polarization solutions.

For the first half of this work, we explored the spatial dynamics when *D_s_* = 0 (i.e. no surface diffusion). Initially we set *k*_1_ = 0 in Eq (1.1) so that there would be no positive feedback. Amplification would arise from the cooperative production of *a* as a function of input (*k*_0_/(1+(*βu*)^−*q*^)). With the parameter *β* = 1, and the average ligand concentration *L_mid_* = 1, we varied the slope of the gradient (*L_slope_*) for four different values of the Hill cooperativity parameter *q*. A maximum polarization of *a_f_*∼2 (*POL*(*a*)∼1 µm^−1^) was achieved. Increasing *q* resulted in better polarization at smaller slopes (i.e. shallower gradients), and thus better amplification (Fig. 2A). We were able to increase polarization beyond *a_f_* = 2 by fine-tuning *β* such that *βL_f_* (*L_f_* is the ligand concentration at the front of the cell) was closer to 1. For *β* = 0.92, *a_f_* = 8 (Fig. 2B).

For a given value of *L_mid_*, it was possible to obtain good polarization for a shallow slope using a high value of *q* and fine-tuning the value of *β*. What happens when we vary *L_mid_* for a fixed *β* and *q*? Good polarization was observed only for a narrow range of concentrations. In Figure 2C, we varied *L_mid_* (for a fixed *L_slope_* relative to *L_mid_*) over a 2-fold range from 1 to 2 for different values of the cooperativity parameter *q*. There was a tradeoff: higher values of *q* produced better polarization, but a reduced range of responsiveness. More importantly, the overall range was quite limited (less than 2-fold), thus indicating a very narrow dynamic range of the polarization response with *k*_1_ = 0.

We added positive feedback by setting *k*_1_>0; *a* acts autocatalytically to stimulate its own production. Within the positive feedback term, there is a Hill cooperativity parameter *h*. Both *k*_1_ and *h* influenced the strength (gain) of the positive feedback. For *k*_1_ = 1 s^−1^, polarization improved for higher values of *h* (Fig. 2D). The increase in polarization was accompanied by the appearance of multiple steady-states (blue lines). We represented these steady-states by an envelope of possible solutions. We then explored different values of *k*_1_ for fixed values of *h*. With *h* = 1, there was no enhanced amplification even for large values of *k*_1_. Thus, substantial amplification required some degree of cooperativity in the positive feedback loop [22]. With *h*≥2, we saw increased maximum polarization for higher values of *k*_1_. Thus, increasing *k*_1_ or *h* resulted in dramatic polarization that was associated with multiple solutions.

When the positive feedback gain was sufficiently large, a decrease in the gradient slope did not cause a decrease in the maximum polarization solution. Indeed, the maximum polarization could be achieved as *L_slope_*→0, indicating the presence of infinite amplification or what has more commonly been termed spontaneous polarization (i.e. polarization in response to an infinitesimal gradient) [11]: *AF*→∞ when *POL*(*L*)→0 and *POL*(*a*)→*C*>0 (Fig. 2E). Interestingly at higher gradient slopes there was actually a slight decline in maximum polarization. In Figure 2E, the envelope of possible solutions is indicated by the region between the solid lines (maximum polarization solution) and dashed lines (minimum polarization solutions).

Plotting *a_f_* versus *L_mid_* revealed a broad dynamic range for the maximum polarization solution spanning at least four orders of magnitude for higher values of *h* (Fig. 2F). At larger values of *L_mid_*, polarization decreased but was still substantial for *h* = 4 and *h* = 8. The decrease was caused by the increased contribution of the input-dependent Hill term at all positions both front and back. In summary, one potential role of positive feedback in biological systems is to increase the amplification and dynamic range of gradient-induced polarization.

### Multiple Steady-States Arise from Positive Feedback

Given that there are multiple steady-states, how can one describe all such solutions? Simulations identify a subset of solutions one by one; analytic methods are needed to determine the range of possible solutions. At steady-state the time derivatives (left-hand side) of the differential Equations (1.1) and (1.2) in Model 1 go to 0, and then one can solve the resulting algebraic equations for *a*: 0 = *f*(*a*, *b*, *z*). The solution must also satisfy the integral constraint imposed by integral control: 0 = *a̅*(*b*)−*a_ss_*. Because multiple values of *b* may satisfy the constraint, one scans for feasible *b*, *b_s_*, and then solves for the roots of the polynomial equation *f*(*a*, *b_s_*, *z*) = 0.

**Figure 3:**
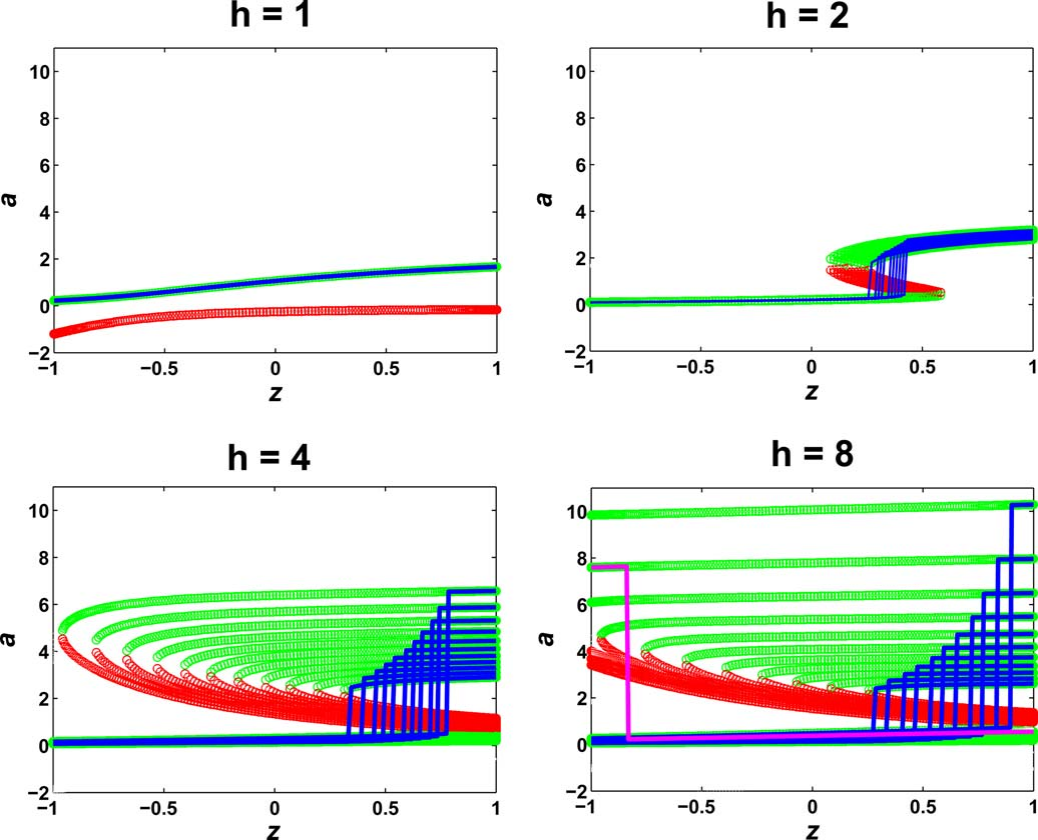
Root curves of steady-state equations define multiple steady-state solutions. The root curves displaying the steady-state solutions of one model for increasing values of *h*. Each curve represents the roots for a particular value of *b* that satisfies the integral constraint; both stable roots (green circles) and unstable roots (red circles) are present. The highest polarized solution for each root curve is traced in blue. For *h* = 8, a reversed polarization solution is shown in magenta, which arises from a “three-tier” root curve that is not contiguous within the dimensions of the cell.

For didactic purposes, we explored a version of the model in which we let *γ* = *γ′*(1/(1+(*βu*)^−*q*^)), *γ*′ = 1 (see Section 2.5 for further description); the essential results did not depend on the particular model. For *k*_1_ = 0, there was a single solution, and we obtained an expression in which *a* is a function of the input-dependent Hill cooperativity term. For *h* = 1 (*k*_1_ = 10 s^−1^), only one value of *b* satisfied the integral constraint, and the resulting quadratic equation in *a* possessed only one positive root. Thus, there was at most a single steady-state, which is shown in Fig. 3. For *h* = 2, there were multiple feasible values of *b* resulting in a family of root curves. The resulting polynomials were cubic, and depending on the parameter values, there could be one or three real roots, which could be stable or unstable. In Figure 3 (*h* = 2) for a given *b_s_*, we observed a lower stable root and an upper stable root and an overlapping region containing two stable roots and one unstable root. One forms a solution by connecting the stable points along the x-axis in a manner that satisfies the integral constraint, crossing between the lower and upper root curves in the overlapping region (blue lines). There were multiple solutions for each root curve given that one can cross between the lower and upper roots multiple times, but typically we were most interested in the solution with the highest polarization value, which is what is drawn in blue. The envelope of solutions represents the highest polarized solutions for each feasible *b*, and thus does not represent all possible solutions.

For values of *h* greater than 2, we solved for the roots numerically using MATLAB. As *h* increased, the plots became more curvy and “S”-shaped with a broader overlap region, and larger upper stable values. In addition, the range of feasible values of *b* increased resulting in more solutions and a broader envelope of polarized solutions. For *h* = 8, the overlap region of some root curves spanned the entire length of the cell. We termed such root curves “three-tier” because the root curve was no longer contiguous within the boundaries of the cell, resulting in three separate segments, the upper and lower stable solutions and the middle unstable solution. Such “three-tier” root curves allowed for reversed polarization solutions in which the intracellular component was concentrated at the wrong end of the cell where the ligand concentration was lower (magenta line; Fig. 3, *h* = *8*); such a situation may arise from flipping the gradient.

We examined these root plots as we varied other parameters. In general, increasing the contribution of the positive feedback to polarization (e.g. increasing *k*_1_, decreasing *L_slope_*, etc.) resulted in more “S” shaped root curves, a broader envelope of possible solutions, and greater maximum polarization. Thus, a more comprehensive picture of the spatial dynamics of the model emerges from the steady-state analysis, which highlights potential tradeoffs.

### Tradeoff between Tracking and Amplification

**Figure 4:**
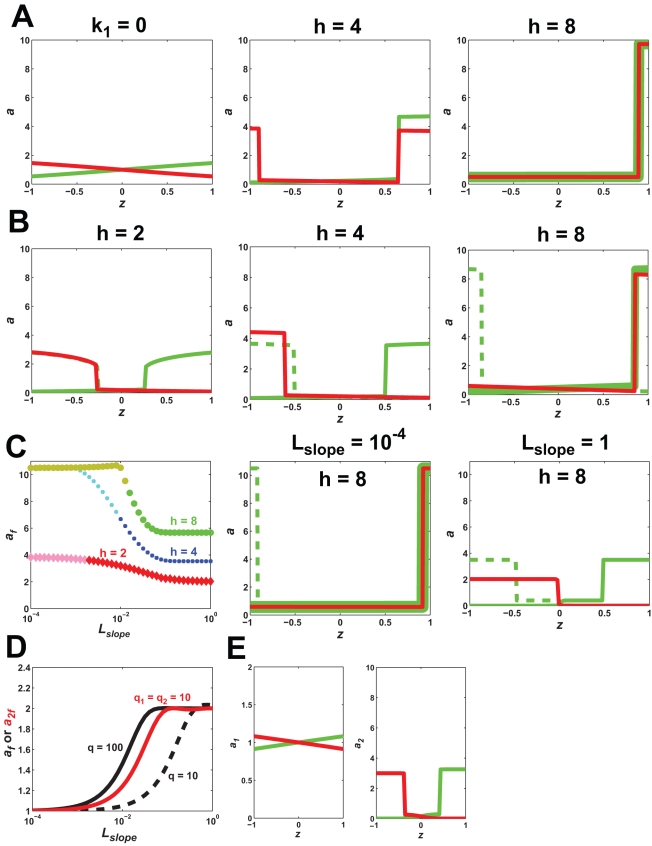
Tracking in standard positive feedback (SF), feedforward/feedback (FF), and multi-stage (MS) models. Simulations (not analytical solutions) determined the polarization in plots in which the gradient direction of the source was switched from the right to the left (*L_mid_* = 1, *L_slope_* = 0.01 µm^−1^). The forward gradient polarization solution is drawn in green and the reverse gradient solution in red. (A) In the standard feedback (SF) model, the no positive feedback case (*k*_1_ = 0) is to the left. For (*k*_1_ = 10 s^−1^, *h* = 4 or 8), the SF model cannot track the directional change. (B) In the feedforward/feedback (FF) model (*k*_1_ = 10 s^−1^, *q* = 100), we observed better tracking but at the expense of the polarization. The dashed green line represents the mirror-image of the forward gradient polarization. (C) The maximum polarization solutions for the FF model as the gradient slope was varied for *h* = 2, 4, 8. For each *h*, at lower slope values, there was a transition denoted by the lighter shading to the presence of “three-tier” roots and higher polarization but reduced tracking. For *h* = 8, the polarization at the shallower slope (*L_slope_* = 10^−4^ µm^−1^) was greater than at the steeper slope (*L_slope_* = 1 µm^−1^), but some tracking was possible only at the steeper slope. (D) The polarization solution for the single-stage (Model 1) model with only cooperativity (*k*_1_ = 0, *q* = 10 or 100) is redrawn in black (solid and dashed, respectively). The multi-stage (MS) Model 2 with only cooperativity is drawn for *q*_1_ = *q*_2_ = 10 (red line). (E) Gradient directional switch in the MS model. There was amplification of the input gradient (*L_slope_* = 10^−3^ µm^−1^) to a steeper gradient of *a*_1_ (*h*_1_ = 1), and as a result, we observed tracking even after the more substantial amplification in the second stage (*h*_2_ = 8).

When the gain (strength) of the positive feedback was high, amplification was substantial when considering the most polarized solution. However, what happens when the gradient is flipped? Biologically, the source of a gradient (e.g. yeast mating partner) may be moving with respect to the sensing cell. We tested the ability of the model to track a 180° change in the gradient direction for different parameter values. In this section, we used simulations to select a single steady-state solution instead of using analytic methods to define all possible solutions. In the case of the pure cooperativity model with no positive feedback (*k*_1_ = 0), tracking was perfect; the polarized distribution of *a* always aligned with the gradient (Fig. 4A, *k*_1_ = 0). Adding positive feedback by increasing *k*_1_ improved polarization, but at the cost of tracking. With *k*_1_ = 10 s^−1^ and *h* = 4, flipping the gradient resulted in polarization that partially tracked the change, and when *h* = 8, the polarized species became stuck in the initial direction and did not track the 180° change in direction at all (Fig. 4A). Thus, there was a tradeoff between amplification and tracking.

A simple explanation is that tracking was impaired because of the presence of multi-stability (multiple steady-states) arising from the positive feedback, including steady-states in which the polarization was not correctly aligned in the same direction as the gradient. Cooperativity alone has a single-steady-state and hence can track perfectly, but without positive feedback, amplification is limited in terms of the magnitude and dynamic range. For moderate levels of positive feedback the polarization can be greater, but tracking was compromised because of the existence of partially polarized solutions that can be reached when the gradient direction was switched. For high levels of positive feedback, there was infinite amplification (spontaneous polarization), but also solutions in which the polarization was reversed with respect to the gradient.

Intuitively, the positive feedback overwhelms any dependence on the current input. As a result, hysteresis can arise in which the polarization depends on the past history of inputs to the cell, as well as the current input, so that tracking is impaired. Thus, positive feedback increases amplification, but also results in multi-stability and the loss of tracking.

### Feedforward/Feedback Coincidence Detection in Positive Feedback Loop Improves Tracking

It would be desirable to obtain a compromise between the potent amplification obtained from high-gain positive feedback with the perfect tracking obtained from pure cooperativity in order to achieve good tracking and polarization under a range of environmental conditions. We developed a modified version of the model that could better balance amplification and tracking. We adjusted the positive feedback term to include a dependence on the input *u*: 
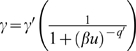
, *γ*′ = 1 and *q*′ = *q*. One can interpret this modification as a type of feedforward/feedback coincidence detection [23] in the positive feedback loop. The result is that the positive feedback term has a dependence on both *a* and *u* (Fig. 1C). The input-dependence of the positive feedback is modulated by the cooperativity parameter *q*′ in the Hill term. Thus, the feedback amplification of *a* has a feedforward component from *u* and a feedback component from *a*, and these must coincide to obtain the best amplification. Biologically, one can implement such a mechanism by the convergence of two signaling pathways, one of which is part of a positive feedback loop.

We tested the feedforward/feedback (FF) model by switching the gradient 180° using the default input values of *L_mid_* = 1 and *L_slope_* = 0.01 µm^−1^. As before, increasing *h* resulted in better polarization, but decreased tracking. Compared to the standard positive feedback model (SF), however, the FF model displayed better tracking, but reduced polarization (Fig. 4B). For *h* = 2, the tracking was nearly perfect whereas in the standard model tracking was impaired for *h* = 2 (data not shown). For *h* = 4, again there was better tracking than the comparable SF model although the fact that the forward and reverse gradient solutions were not the same indicates multi-stability, which was associated with some loss of tracking. For *h* = 8, there was no tracking as was observed with the SF model.

Like the SF model, the FF model showed constant polarization and hence infinite amplification (spontaneous polarization) as the gradient slope approached 0. Interestingly, at steeper slopes the polarization actually decreased. We hypothesized that at the higher slopes there was stronger input-dependence of the polarization and hence reduced amplification, but better tracking. To check this possibility we examined the results from a 180° directional change for a small gradient slope and for a large gradient slope. As expected, when *L_slope_* = 0.0001 µm^−1^, there was no tracking but good polarization, whereas when *L_slope_* = 1 µm^−1^, there was some tracking but reduced polarization (Fig. 4C). In this figure, we also indicated the transition to the appearance of “three-tier” root curves described previously that can give rise to reversed polarization solutions. This transition occurred as *L_slope_* decreased and the polarization jumped to a higher value. Thus, decreasing the input-dependence of the positive feedback by reducing the gradient slope, results in an increase in polarization but a loss in tracking.

### Multi-Stage Amplification Can Improve Amplification or Tracking

In Model 1, the amplification resulting in polarization is achieved through the dynamics (i.e. positive feedback and cooperativity) of one species. We explored a model containing two polarized species in a cascade resulting in two amplification stages. The first polarized species *a*_1_ serves as the input to the second stage which gives rise to the polarization of the second species *a*_2_. We essentially duplicated Model 1 to form Model 2:
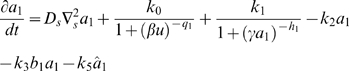
(2.1)

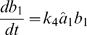
(2.2)

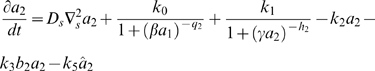
(2.3)

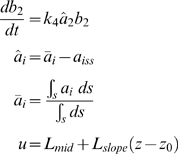
(2.4)One advantage of two stages is that amplification can be combined to achieve a larger net amplification. For example, a single reaction may produce a limited amount of cooperativity. Cascading two cooperative reactions together can result in a higher total cooperativity. In Model 2, we let *k*_1_ = 0 so that there was no positive feedback. In Figure 4D we redrew the amplification of Model 1 using *q* = 10 or *q* = 100. It may be difficult for a single reaction to produce a Hill cooperativity of 100. However, when we cascaded two reactions with *q*_1_ = *q*_2_ = 10, then the final cooperativity approached that of 100. Indeed, it is common practice in engineering to link together amplifiers to attain greater amplification [24].

A second advantage for two stages is better tracking. The first stage can amplify the external gradient so that the input to the second stage is steeper than the original input. In Figure 4B, we observed that for *h* = 8 and *L_slope_* = 0.01 µm^−1^, there was no tracking (but excellent polarization) whereas at *L_slope_* = 0.1 µm^−1^, the polarization was reduced but the tracking was better (data not shown). We constructed a multi-stage model in which the first-stage amplification was approximately 100 (*AF*∼100) so that the initial ligand slope *L_slope_* = 0.001 µm^−1^ was transformed into a slope of *a*_1_ that was approximately 0.1 µm^−1^. In the second stage (*h* = 8), there was some tracking of the directional change by the polarized species *a*_2_ because of the steeper input gradient of *a*_1_ (Fig. 4E).

A third advantage, which is not investigated here, is that having two stages can produce a broader dynamic range. The input to the first stage produces a normalized input to the second stage. Thus, the negative feedback in the first stage effectively shrinks the dynamic range being fed into the second stage. From a biological standpoint, one can propose that the cascaded arrangement of the heterotrimeric and the Cdc42p G-protein cycles results in multiple amplification stages, and we exploited this concept in our model of yeast cell polarization.

### Polarization and Tracking in 2D Simulations

To this point, the simulations and analyses employed an axisymmetric 1D geometry so that it was only possible to change the direction of the gradient 180°. A greater challenge would be responding to more subtle directional changes. To this end, we constructed a two-dimensional (2D) model of the cell, which was represented as a circle, so that the gradient could be applied in any direction on the circle. Using this new model, cells were polarized with an initial gradient and then the direction of the gradient was changed. After the shift, we compared the direction of the polarization with the direction of the gradient as a measure of tracking.

**Figure 5:**
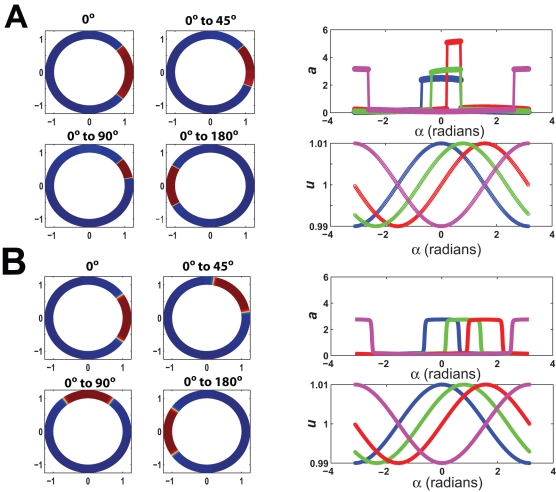
Tracking changes in gradient direction using simulations of two-dimensional polarization model. The gradient direction (*L_mid_* = 1, *L_slope_* = 0.01 µm^−1^) was initially at 0° (3 o'clock position), and then shifted 45°, 90° or 180° in the counterclockwise direction. The response of the cell was depicted either on a disk (left figures) in which the value of *a* is color-coded (dark red = high, blue = low) or in a perimeter plot in which x-axis describes the radial position and the y-axis the value of *a* or the input *u*. In the perimeter plot, there is a curve for each new gradient direction (blue = 0°, green = 45°, red = 90°, magenta = 180°). (A) Results of gradient directional change in FF model (*h* = 4) without diffusion. (B) Results of gradient directional change in FF model (*h* = 4) with diffusion (*D_s_* = 0.001 µm^2^/s).

Model 1 with only cooperativity (*k*_1_ = 0) displayed perfect tracking for directional changes of 180°, 90°, and 45° as expected since there is a single solution (data not shown). For the feedforward/feedback model described above (*h* = 4, *q* = 100) we observed tracking of the 180° degree directional change (although a different polarization solution), but the polarization was not aligned with the gradient for the 90° and 45° changes (Fig. 5A). Thus, the 2D simulations offer a more stringent test of tracking, and more accurately reflects the conditions of a cell confronted with a shifting gradient.

### Surface Diffusion in Membrane Reduces Number of Steady-States

Diffusion can exert a profound effect on spatial dynamics [4]. The small size of the yeast cell and the fast rate of diffusion in the cytoplasm for a freely diffusible protein caused us to focus on lateral surface diffusion in the membrane. Proteins in the membrane are able to diffuse laterally in the plane of the membrane [25]. One would expect surface diffusion would dampen cell polarization by allowing proteins to diffuse away fr**7**om sharp concentration peaks. However, what would happen to the multiple steady-states in the presence of lateral diffusion? Would the envelope of solutions become less polarized or would the envelope be modified in some way?

**Figure 6:**
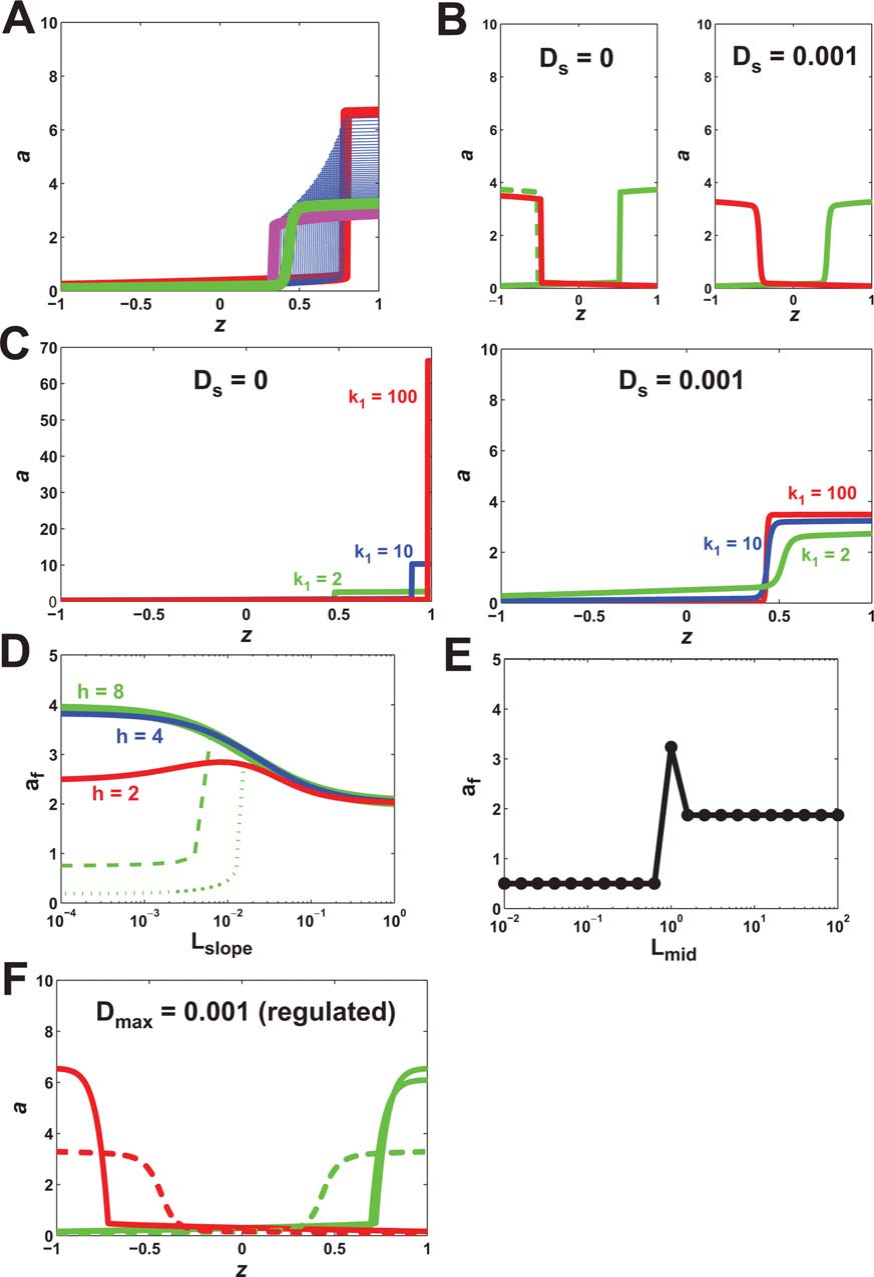
Lateral surface diffusion enhances the robustness of polarization. In the gradient, *L_mid_* = 1 and *L_slope_* = 0.1 µm^−1^. (A) Surface diffusion selects a single solution among multiple steady-states. The polarization envelope (blue lines) is shown for the FF model (*k*_1_ = 10 s^−1^, *h* = 8) with no diffusion (red line, maximum polarization; purple line, minimum polarization). The single steady-state solution in the presence of diffusion (*D_s_* = 0.001 µm^2^/s), (thick green line). (B) Diffusion improves tracking. A 180° directional change is shown with and without diffusion (green line represents initial polarization, red line represents reversed polarization, dashed green line represents the reversed polarization solution symmetric to initial polarization). The overlap between the dashed green and red lines in the presence of diffusion suggests a single steady-state solution and perfect tracking. (C) Presence of diffusion ensures that polarization is robust to variations in the positive feedback. When *D_s_* = 0, increasing *k*_1_ results in a dramatic increase in the maximum polarization solution. In the presence of diffusion there is almost no change in polarization for larger *k*_1_ (note reduced scale of y-axis in right graph). (D) Polarization as a function of *L_slope_* (*L_mid_* = 1) in the FF model with diffusion (*D_s_* = 0.001 µm^2^/s). For *h* = 8, there were two additional solutions for smaller *L_slope_* values: an unpolarized *b* = 0 solution (dashed green line) and a reversed polarized solution (dotted green line). (E) Polarization as a function of *L_mid_*, *L_slope_* = (0.01×*L_mid_*) µm^−1^, in the FF model with diffusion (*h* = 8). (F) Regulating diffusion can produce stronger polarization. Using the regulated diffusion term described in the text, enhanced polarization seen compared to constant diffusion (dashed lines). There were two forward polarization solutions, and both gave the same reversed solution when the gradient was flipped.

Introducing diffusion caused the envelope of solutions to collapse to a single solution. In Figure 6A, we overlay the single solution with *D_s_* = 0.001 µm^2^/s among the envelope of steady-state solutions with *D_s_* = 0; the diffusion solution is positioned toward the rear of the envelope. It is important to note that the presence of diffusion prevents analytic solutions to the model. Instead, we employed exhaustive simulations from a wide variety of initial conditions to identify any stable steady-states, but simulations cannot guarantee that we have found all solutions.

However, the response of the FF model with diffusion to changes in gradient direction also argues for a single steady-state. When *D_s_* = 0, a 180° change in direction resulted in a polarized solution different from the initial polarization. When *D_s_* = 0.001 µm^2^/s, the initial and final polarization were identical (Fig. 6B). A more stringent test was with the 2D models. In the presence of diffusion, the cell could accurately track directional changes in the gradient of 90° and 45° (Fig. 5B). Thus, in biological systems, lateral diffusion in the membrane may play an important role in preventing multi-stability during polarization.

**Figure 7:**
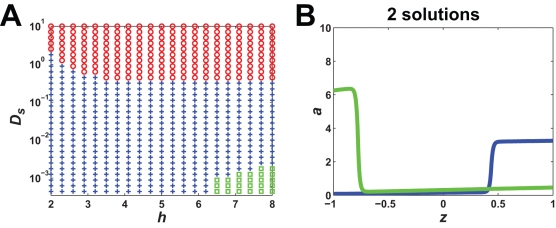
Bifurcation diagram of model behavior as a function of the parameters *D_s_* (diffusion) and *h* (positive feedback). (A) This plot was constructed by exhaustive simulation of the FF model (*k*_1_ = 10 s^−1^; *L_mid_* = 1, *L_slope_* = 0.01 µm^−1^) over a range of parameter pair values (*D_s_*, *h*). Three behavior classes were observed. (1) One steady-state solution (blue, +). (2) Two solutions (green, square). (3) Limit-cycle oscillations (red, circle). (B) An example of the two solutions class in which there is a forward (blue) and a reversed (green) polarization solution to a right-to-left gradient.

Polarization in the presence of diffusion for the FF model increased when the gradient slope decreased (Fig. 6D) just as it did in the absence of diffusion (Fig. 4C). Again, we interpret this result in terms of the input-dependence of the polarization. Furthermore, polarization maintained a constant value even as *L_slope_*→0 indicative of the infinite amplification (spontaneous polarization) that was observed in the model without diffusion. For the *h* = 8 case, decreasing the slope led to two additional solutions; one is the unpolarized solution with *b* = 0 and the other is a reversed polarization solution (Fig. 7B). Thus, at high levels of positive feedback gain, multiple steady-state solutions could arise with diffusion, but there was no envelope of solutions. Varying *L_mid_* showed a peak at *L_mid_* = 1 (Fig. 6E). When *L_mid_*>1, there was a modestly polarized solution (*a_f_*∼2). For *L_mid_*<1, there was not sufficient activation to achieve polarization. The degree of polarization was much more modest when compared to the no diffusion case, but the dynamic range was still broad.

### Surface Diffusion Limits Extent of Polarization in a Robust Fashion

In the absence of surface diffusion, increasing the positive feedback gain increased the maximum possible polarization among the multiple steady-state solutions. Introducing membrane diffusion prevented the more extreme polarization states from being reached and reduced the number of steady-states. Indeed, one would expect diffusion to counteract the positive feedback concentrating components at the front.

Surprisingly, the presence of surface diffusion also caused the degree of polarization to become robust to changes in the gain of the positive feedback. In the *D_s_* = 0 case, as we increased *k*_1_, we dramatically increased the maximum polarized solution (Fig. 6C). For *D_s_* = 0.001 µm^2^/s, increasing *k*_1_ had only a modest effect on the maximum polarized solution. Diffusion pushed the polarization back toward the least polarized solution in the envelope of steady-state solutions that exist in the absence of diffusion.

More generally, we found that when *D_s_*>0 the extent of polarization became relatively insensitive to changes in a wide range of internal and external parameters (e.g. *L_slope_*, *L_mid_*, *k*_1_, *h*, etc.). Thus, surface diffusion adds robustness to polarization. From a biological standpoint, it may be beneficial to cells to have consistent polarization under different conditions. For example, yeast cells may not want the width of the mating projections to be too sensitive to variations in the concentration or gradient slope of mating pheromone.

### Regulating Diffusion Enhances Polarization

The presence of lateral diffusion in the model, prevents the appearance of highly polarized states in Model 1. Yet, there are circumstances when a cell will want a particular protein localized to a narrow region at the front [3]. One possibility is to regulate the diffusion coefficient in some manner. We postulated that the diffusion coefficient could depend on *a* and developed the following functional form: 
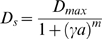
, where 
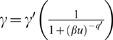
 and we let *q* = *q*′ = 100 and *m* = 8. This term effectively creates a diffusion barrier so that positions in the cell where *a* is high (front), *D_s_*→*D*_max_, whereas at positions where *a* is small *D_s_*→0.

For *D*_max_ = 0.001 µm^2^/s, we examined polarization in the high positive feedback case (*k*_1_ = 10 s^−1^, *h* = 8). Compared to unregulated diffusion, we observed more pronounced polarization which peaked at *a_f_*∼9. There was also the appearance of more than one steady-state, but the number of steady-states was smaller than in the *D_s_* = 0 case; only two steady-states were found from extensive simulations (Fig. 6F). Consistent with the fewer solutions, tracking of a 180° change in gradient direction was good. Thus, regulating diffusion balances polarization with tracking, and biologically this additional level of modulation could be important in optimizing the polarization response.

### Increasing Diffusion Promotes Oscillations

Increasing the positive feedback gain results in multi-stability and extremely polarized solutions. Increasing diffusion reduces multi-stability and polarization. What happens with high levels of both positive feedback and diffusion?

Using simulations, we constructed a bifurcation diagram summarizing the dynamical behaviors for different values of *h* and *D_s_* in a version of the FF model possessing integral but not proportional negative feedback (i.e. *k*_5_ = 0). In Figure 7A, we explored values of *D_s_* from 10^−3^ to 10 µm^2^/s and values of *h* from 2 to 8. For lower values of *h* and *D_s_*, we observed a single steady-state solution. Increasing *h* with small *D_s_* resulted in two steady-state solutions; interestingly, the second solution was a reversed solution in which the polarization was at the rear of the cell relative to the gradient (Fig. 7B). Increasing *D_s_* and to a lesser extent increasing *h* produced limit-cycle oscillations. Thus, there is a danger of instability for biological systems when diffusion and positive feedback are too high. It is important to emphasize that these results were derived from exhaustive simulations, and thus we cannot exclude the possibility that additional solutions exist.

### Constructing a New Model of Yeast Cell Polarization Induced by Mating Pheromone Gradients

This research was motivated by an interest in yeast cell polarization, and one of the primary goals was to apply the insights gained from the generic models to models more specific to yeast. Our past efforts modeling yeast cell polarization [19] were hampered by the complexity of the model. The work described above helped us to understand the model behavior and make improvements. This model was based on the spatial dynamics of the heterotrimeric and Cdc42p G-protein cycles. Receptor (R) binds ligand (L) and becomes activated (RL). Activated receptor converts heterotrimeric G-protein (G) into activated α-subunit (Ga) and free Gβγ (Gbg). All of these species are on the membrane. The connection between the two cycles is the fact that free Gβγ recruits cytoplasmic Cdc24p to the membrane. Membrane-bound Cdc24p (C24m) activates Cdc42p. Activated Cdc42p (C42a) recruits the scaffold protein Bem1p (B1) to the membrane. Finally, a positive feedback loop is created because membrane-bound Bem1p can bind and recruit Cdc24p to the membrane. All components residing on the membrane were subject to the same lateral diffusion. It is important to note that the model lacks an explicit consideration of ligand-stimulated endocytosis and polarized synthesis which are known to be crucial for many aspects of cell polarity [26], [27] and are the subjects of future research. For this work, we focused on the fast positive feedback loop mediated by Bem1p [28].

The connection between the yeast model and the generic model (Model 1) is best seen in equation describing the dynamics of membrane-bound, active Cdc24p (C24m, Eq. (3.5)). There, recruitment of Cdc24p to the membrane depends on a cooperative term that is a function of Gβγ, (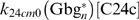), and a positive feedback term, (*k*_24*cm*1_(*B*1^*^)[C24c]), that depends on Bem1p which in turn is a function of active Cdc42p and hence active Cdc24p. We made two important modifications to the previous model. First, we added a negative feedback loop for better regulation. The loop includes the protein kinase Cla4p which is activated by Cdc42p and which phosphorylates and inhibits Cdc24p resulting in negative feedback [29]. Second, there is a feedforward/feedback coincidence detection term in the positive feedback loop for better tracking that involves Gβγ. The input to the model was a gradient of the mating pheromone alpha-factor; the output was active Cdc42p ([C42a]).

The first four equations (3.1 to 3.4) describe the spatial dynamics of the heterotrimeric G-protein cycle, and the next five equations (3.5 to 3.9) describe the spatial dynamics of the Cdc42p G-protein cycle. The two-stage structure of the model was important for extending its dynamic range.
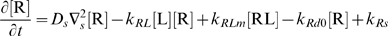
(3.1)

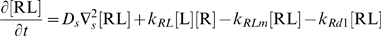
(3.2)

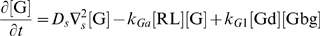
(3.3)

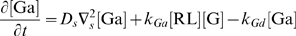
(3.4)

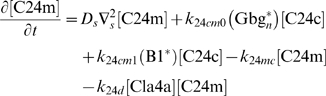
(3.5)

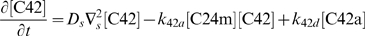
(3.6)

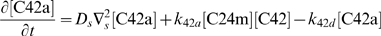
(3.7)

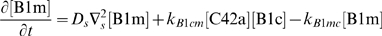
(3.8)

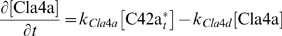
(3.9)A more detailed description of the model is in the Appendix S1 (Supporting Information).

### Modeling the Effect of Surface Diffusion on the Robustness of Yeast Cell Polarization

**Figure 8:**
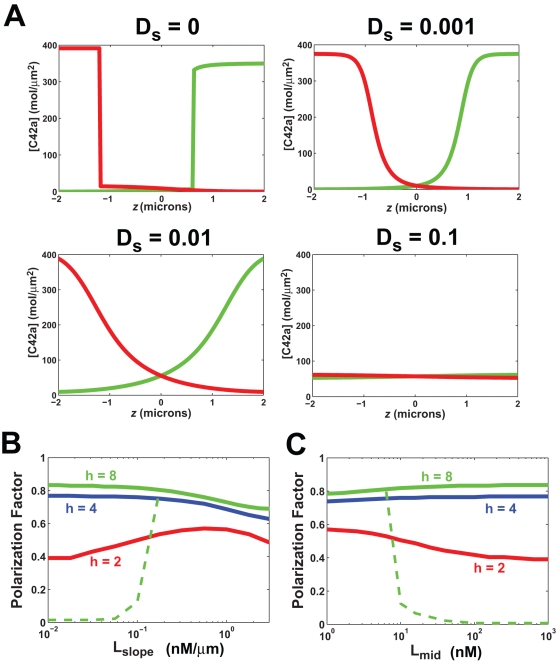
Simulations of spatial model of yeast cell polarization. (A) Effect of lateral surface diffusion on mating factor induced polarization in yeast cell model. The input was an alpha-factor gradient (*L_mid_* = 10 nM and *L_slope_* = 1 nM/µm) and the output was the steady-state concentration of active Cdc42p ([C42a]), which was plotted along the axial length of the cell. The results were for different surface diffusion coefficients. (B) Polarization in yeast model as a function of *L_slope_* (*L_mid_* = 10 nM) in the yeast model with diffusion (*D_s_* = 0.001 µm^2^/s). Polarization is described in terms of the polarization factor (maximum polarization = 1; unpolarized = 0; *PF* = 0.8 corresponds to *a_f_*∼5; *PF* = 0.5 corresponds to *a_f_*∼2). Three values of *h* were examined: *h* = 2 (red), *h* = 4 (blue), *h* = 8 (green). For *h* = 8, there was an additional unpolarized solution (dashed green line) for smaller *L_slope_* values. (C) Polarization as a function of *L_mid_*, *L_slope_* = (0.01×*L_mid_*) µm^−1^, in the yeast model with diffusion for three values of *h*.

Using the yeast model, we wished to estimate the range of surface diffusion coefficients that would permit robust polarization in yeast cells in response to mating pheromone. When *D_s_* = 0, there was good polarization, but also multi-stability, which was manifested when we reversed the gradient and an alternative polarized solution appeared that was not identical to the initial polarization (Fig. 8). Adding lateral diffusion with *D_s_* = 0.001 µm^2^/s resulted in a single steady-state with perfect tracking for the input conditions. Increasing *D_s_* ten-fold (*D_s_* = 0.01 µm^2^/s) maintained a comparable level of polarization, although the shape of the distribution was altered. For *D_s_* = 0.1 µm^2^/s, polarization was abolished. Thus, in this model, the highest range of *D_s_* that produced good polarization was between 0.01 and 0.001 µm^2^/s. In a previous section, we demonstrated that larger values of diffusion were associated with better tracking and a reduced likelihood of multi-stability. In yeast, the measured value of *D_s_* was 0.0025 µm^2^/s [30], and so our simulations suggest that membrane fluidity in yeast has been tuned for robust polarization.

We also examined the dynamic range and sensitivity to shallow gradients of the yeast model with *D_s_* = 0.001 µm^2^/s. Interestingly, the yeast model displayed similar qualitative behavior to the generic FF model with diffusion (compare Figs. 6 and 8). For *h* = 4 and *h* = 8, polarization in the yeast model was observed even at relative slopes less than 0.01 µm^−1^ (Fig. 8B). For *h* = 8, there was an additional steady-state solution in which the cell was unpolarized (Fig. 8B, green dashed line). The dynamic range of gradient-sensing and polarization was excellent in the yeast model for *h* = 4 and *h* = 8, extending beyond *L_mid_* = 1000 nM (Fig. 8C). Polarization was greater than in the generic model in part because of the two-stage (two-cycle) structure of the yeast model. For *h* = 2, polarization declined at higher values of *L_mid_*, and for *h* = 8, there was an additional unpolarized solution; *h* = 4 represented a compromise. Finally, we ran 2D simulations of the yeast model (*h* = 4) which exhibited good tracking to changes in the gradient direction (data not shown) again resembling the generic FF model with diffusion. Thus, the yeast model could show robust performance with a balance of amplification, dynamic range, and tracking. It should be noted that recent experiments have demonstrated that yeast cells can sense pheromone gradients possessing relative slopes as shallow as 0.001 µm^−1^ and at concentrations as high as 1000 nM (T.I. Moore, C.S. Chou, Q. Nie, N. L. Jeon and T.-M. Yi, submitted), and so this modeling can help serve as a framework for future more realistic models that contain more detailed reaction mechanisms.

## Discussion

In this paper, we investigated the spatial dynamics of cell polarization induced by chemical gradients focusing on the tradeoff between amplification and tracking and on the impact of lateral surface diffusion on polarization. Previous work has noted this tradeoff, but we wished to explore its nature in greater depth by using a generic model and steady-state analysis. A highly cooperative response to the input resulted in good tracking of a moving signal source, but amplification to produce potent polarization was limited to a very narrow range of concentrations. Adding high-gain positive feedback resulted in strong amplification over a broad range of concentrations, but tracking was poor.

Intuitively, one can understand this tradeoff in terms of the input-dependence of the amplification. High input-dependence is necessary for tracking, but then weaker inputs (i.e. shallow gradients) will not be amplified as well. On the other hand, low input-dependence results in good amplification regardless of the input strength, but then tracking a directional change in the input becomes difficult (i.e. polarization becomes stuck).

An important technical tool was the application of steady-state analysis to the model. The positive feedback led to multiple steady-states, which we were able to describe by analytical solutions to the model equations. We could then see the connection between increased positive feedback, a larger envelope of steady-states, amplification that was not input-dependent, and the loss of tracking. With a single steady-state, tracking is perfect, whereas with multiple steady-states, there could exist solutions in which the polarization is not aligned in the same direction as the gradient.

Living systems evolve to find the appropriate balance for this tradeoff in a given environment. There must be sufficient amplification to induce the proper polarization for gradients typically encountered. Likewise, tracking is a significant consideration if the signal source is expected to move. In the context of the yeast mating response, there must be sufficient polarization to form a mating projection over a range of background pheromone concentrations, which may vary according to the number and proximity of mating partners, and at the same time, the ability to redirect the projection if the partner moves or mates with another cell. We constructed a modified model in which feedforward/feedback coincidence detection improved tracking with some loss in dynamic range. Tracking performance could be further improved using multi-stage amplification to split the amplification.

The presence of lateral surface diffusion significantly altered polarization behavior. First, at low diffusion coefficients, it collapsed the multiple solutions to fewer solutions, and in certain cases, to a single solution. As a result, tracking was improved, but the extent of polarization was reduced. When combined with the feedfoward/feedback coincidence detection, low levels of lateral diffusion produced perfect tracking over a range of input gradient conditions. A second effect of lateral diffusion was that the degree of polarization was quite robust to changes in the parameter values. It may be advantageous to cells that polarization is robust to variations in internal and external conditions. Third, high levels of diffusion coupled to high positive feedback resulted in oscillations. Together, these results argue that maintaining the proper level of diffusion in the membrane is critical for robust polarization. It is important to mention that there is a concern that some of these conclusions may depend on the particular model structure. Although we attempted to formulate a “general” generic model structure, further research is needed to address this concern.

We took the lessons from the simple model and incorporated them into a more complex model of yeast polarization. In particular, we implemented feedforward/feedback coincidence detection via Gβγ influencing the Cdc24p-Cdc42p-Bem1p positive feedback loop, and also implemented negative feedback regulation of Cdc24p. The resulting model exhibited good polarization, gradient sensitivity, and dynamic range. In the future, we plan to improve the model by adding multi-stage amplification that takes advantage of polarized synthesis and endocytosis of the pathway components. In addition, we would like to add more mechanistic elements and evaluate the robustness of the models.

From this research, certain predictions and explanations arise. First, we expect the cellular polarization apparatus to contain elements that generate both cooperativity and positive feedback, and the amount of each depends on the appropriate amplification/tracking balance suitable for the cell in its natural environment. Second, we identify feedforward/feedback coincidence detection and multi-stage feedback as important strategies for improved tracking ability of cells. Third, we demonstrated that lateral surface diffusion contributes significantly to the robustness of polarization, and predict that this diffusion will be carefully regulated. Fourth, we used simulations of yeast cells to show that proper polarization was achieved using values of the diffusion coefficient between 0.01 µm^2^/s and 0.001 µm^2^/s, and indeed Valdez-Taubas and Pelham [30] have measured a value of 0.0025 µm^2^/s.

In the future, we will address additional robustness issues relating to cell polarization induced by spatial gradients. Foremost among these is handling the presence of noise. Stochastic noise arises from fluctuations in the gradient, Brownian motion of the cell, the random nature of the discrete binding events between ligand and receptor [31], etc. These stochastic variations must be distinguished from more meaningful changes in the gradient signal such as a directional change caused by the movement of the signal source. Separating signal from noise is a classic problem in engineering and requires some type of noise filtering [32]. In addition to external noise, there is internal noise arising from variations in the levels and functioning of system components. Regulatory systems must exist to ensure robust polarization in the presence of this internal uncertainty. Furthermore, it is important to investigate different control strategies for improving robustness. For example, an adaptive control strategy involving the self-tuning of key system parameters could make the system more robust to both internal and external variations. Finally, we would like to connect this research more closely to the biology of yeast cell gradient-sensing and polarization.

## Materials and Methods

### Simulations

The surface diffusion of a quantity *W* on an axisymmetric surface in a three dimensional space has the following expression:
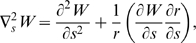
(4)where 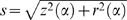 is the arclength of the cell membrane. Consequently, the equations in Model 1 becomes one-dimensional in terms of the parameterization variable α$, even though the cell is a three dimensional axisymmetric ellipsoid.

For a system in the two-dimensional space, in which the cell surface is a curve, the expression of the surface diffusion of a quantity *W* becomes
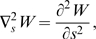
(5)where 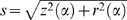 is the arclength of the cell membrane.

Numerical discretizations of each variable on the cell membrane were carried out in α for both cases. All spatial derivatives in the equations were approximated using a second-order finite difference discretization. The temporal discretization was carried out using a fourth order Adams-Moulton predicator-corrector method.

In a typical simulation, the number of grid points in space was 200 with a time-step of 5×10^−4^ s. We tested a range of grid and time-step sizes to assure convergence of the simulations. The simulations in this paper were well-resolved with the above discretization.

### Steady-State Analysis

Without diffusion, the steady state equations 1.1 and 1.2 of Model 1 have a simple form,
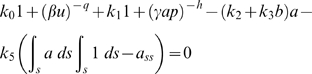
(6)

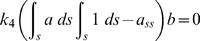
(7)By rewriting Eq. (6) and eliminating the zero solution *b* = 0 in Eq. (7), the steady state system consists of a polynomial equation of *a*, with at most *h*+1 roots, and an equation for the integral control of *a*. The system was solved using the MATLAB polynomial solver ‘ROOT’. We carried out linear stability analysis around each steady-state. We selected the stable steady-state solutions satisfying the integral control equation.

For the system with surface diffusion, a nonlinear Gauss-Seidel iteration procedure [33] was used for the simulations.

### Performing *L_slope_* and *L_mid_* Scans in Models Containing Diffusion

We calculated the polarization as a function of *L_slope_* and *L_mid_* in the models containing diffusion by running a series of simulations. In the *L_slope_* scan, we fixed *L_mid_* and scanned through a series of *L_slope_* values evenly distributed on a log scale. We first scanned from lower *L_slope_* values to higher values. At each succeeding scan point, the initial values were taken as the steady-state computed at the previous scan point. The second scan started with the highest *L_slope_* value and proceeded backwards. The *L_mid_* scans were performed in an analogous manner.

## Supporting Information

Appendix S1 (0.20 MB PDF)
