# Supplementary material for: Modeling Robustness Tradeoffs in Yeast Cell Polarization Induced by Spatial Gradients
Source: PLoS One. 2008 Sep 1;3(9):e3103. doi: 10.1371/journal.pone.0003103 (PMC3021495; doi:10.1371/journal.pone.0003103)
Supplement: Appendix S1 — (0.20 MB PDF) [file pone.0003103.s001.pdf]

APPENDIX S1: Model of Pheromone-Induced Yeast Cell Polarization

We updated a previous model [19] of yeast cell polarization using insights gained from this study. This model was based on the spatial dynamics of the heterotrimeric and Cdc42p G-protein cycles. Receptor (R) binds ligand (L) and becomes activated (RL). Activated receptor converts heterotrimeric G-protein (G) into activated α-subunit (Ga) and free Gβγ (Gbg). All of these species are on the membrane. The connection between the two cycles is the fact that free Gβγ recruits cytoplasmic Cdc24p to the membrane. Membrane-bound Cdc24p (C24m) activates Cdc42p. Activated Cdc42p (C42a) recruits the scaffold protein Bem1p (B1) to the membrane. Finally, a positive feedback loop is created because membrane-bound Bem1p can bind and recruit Cdc24p to the membrane.

The connection between the yeast model and the generic model (Model 1) is best seen in Equation 4 of the yeast model, describing the dynamics of membrane-bound, active Cdc24p. There, recruitment of Cdc24p to the membrane depends on a cooperative term that is a function of Gβγ, ($k_{24cm0}(\mathrm{Gbg}_n^*)[\mathrm{C24c}]$), and a positive feedback term, ($k_{24cm1}(\mathrm{B1}^*)[\mathrm{C24c}]$), that depends on Bem1p which in turn is a function of active Cdc42p and hence active Cdc24p.

We made two important modifications to the previous model. First, we added a negative feedback loop for better regulation. The loop includes the protein kinase Cla4p which is activated by Cdc42p and which phosphorylates and inhibits Cdc24p resulting in negative feedback [29]. Second, there is a feedforward/feedback coincidence detection term in the positive feedback loop for better tracking. We changed the *B1** term from $\left(\frac{B1_t^*}{1+(\gamma[\mathrm{B1m}])^{-h}}\right)$ to $\left(\frac{B1_t^*}{1+(\gamma Gbg_n^*[\mathrm{B1m}])^{-h}}\right)$ where now Gβγ (the output of the heterotrimeric G-protein cycle and the input to the Cdc42 cycle) influences the positive feedback via

two terms: the cooperative input Hill-term and the positive feedback term. Biologically, the model hypothesizes that Gβγ directly modulates the positive feedback loop presumably through direct and indirect protein-protein interactions with Bem1p, Cdc24p, and Cdc42p. For example, Gβγ is known to bind to Ste20p which in turn binds Bem1p and Cdc42p [34].

$$\frac{\partial[\mathrm{R}]}{\partial t} = D_s\nabla_s^2[\mathrm{R}] - k_{RL}[\mathrm{L}][\mathrm{R}] + k_{RLm}[\mathrm{RL}] - k_{Rd0}[\mathrm{R}] + k_{Rs} \quad (1)$$

$$\frac{\partial[\mathrm{RL}]}{\partial t} = D_s\nabla_s^2[\mathrm{RL}] + k_{RL}[\mathrm{L}][\mathrm{R}] - k_{RLm}[\mathrm{RL}] - k_{Rd1}[\mathrm{RL}] \quad (2)$$

$$\frac{\partial[\mathrm{G}]}{\partial t} = D_s\nabla_s^2[\mathrm{G}] - k_{Ga}[\mathrm{RL}][\mathrm{G}] + k_{G1}[\mathrm{Gd}][\mathrm{Gbg}] \quad (3)$$

$$\frac{\partial[\mathrm{Ga}]}{\partial t} = D_s\nabla_s^2[\mathrm{Ga}] + k_{Ga}[\mathrm{RL}][\mathrm{G}] - k_{Gd}[\mathrm{Ga}] \quad (4)$$

$$\frac{\partial[\mathrm{C24m}]}{\partial t} = D_s\nabla_s^2[\mathrm{C24m}] + k_{24cm0}(\mathrm{Gbg}_n^*)[\mathrm{C24c}] + k_{24cm1}(\mathrm{B1}^*)[\mathrm{C24c}] - k_{24mc}[\mathrm{C24m}] - k_{24d}[\mathrm{Cla4a}][\mathrm{C24m}] \quad (5)$$

$$\frac{\partial[\mathrm{C42}]}{\partial t} = D_s\nabla_s^2[\mathrm{C42}] - k_{42a}[\mathrm{C24m}][\mathrm{C42}] + k_{42d}[\mathrm{C42a}] \quad (6)$$

$$\frac{\partial[\mathrm{C42a}]}{\partial t} = D_s\nabla_s^2[\mathrm{C42a}] + k_{42a}[\mathrm{C24m}][\mathrm{C42}] - k_{42d}[\mathrm{C42a}] \quad (7)$$

$$\frac{\partial[\mathrm{B1m}]}{\partial t} = D_s\nabla_s^2[\mathrm{B1m}] + k_{B1cm}[\mathrm{C42a}][\mathrm{B1c}] - k_{B1mc}[\mathrm{B1m}] \quad (8)$$

$$\frac{\partial[\mathrm{Cla4a}]}{\partial t} = k_{Cla4a}[\mathrm{C42a}_t^*] - k_{Cla4d}[\mathrm{Cla4a}] \quad (9)$$

$$Gbg_n^* = \frac{R}{1 + (\delta(Gbg_n))^{-q}}, \quad (10)$$

where $\delta = SA/\int_S (Gbg_n)ds$, and $q = 100$, $R = 1$.

$$B1^* = \frac{B1_t^*}{1 + (\gamma Gbg_n^*[\mathrm{B1m}])^{-h}}, \quad (11)$$

where $B1_t^* = \int_S [\mathrm{B1m}]ds/SA$; $\gamma = SA/(2\int_S [\mathrm{B1m}]ds)$. $SA = \int_S ds$ is the surface area of the cell, and $h = 8$.

$$\mathrm{C42a}_t^* = \frac{\int_S [\mathrm{C42a}]\,ds}{SA} \qquad (12)$$

The initial conditions and conservation equations are as follows. We may assume that [C42], [R], and [G] are equally distributed along the surface with a total amount of $C42_t$, R_t, and G_t, respectively.

$[\mathrm{R}]_0 = R_t/SA,\ R_t = 10,000$ molecules/cell,
$[\mathrm{G}]_0 = G_t/SA,\ G_t = 10,000$ molecules/cell,
$[\mathrm{C42}]_0 = C42_t/SA,\ C42_t = 10,000$ molecules/cell,
$[\mathrm{RL}]_0 = 0,\ [\mathrm{Ga}]_0 = 0,\ [\mathrm{C24m}]_0 = 0,\ [\mathrm{C42a}]_0 = 0,\ [\mathrm{B1m}]_0 = 0.$

$[\mathrm{Gd}] = [\mathrm{G}]_0 - [\mathrm{G}] - [\mathrm{Ga}],$
$[\mathrm{Gbg}] = [\mathrm{G}]_0 - [\mathrm{G}],$
$Gbg_n = [\mathrm{Gbg}]/\mathrm{G}_0,$
$V \cdot [\mathrm{C24c}] = C24_t - \int_S [\mathrm{C24m}]ds,\ C24_t = 2000$ molecules/cell,
$V \cdot [\mathrm{B1c}] = B1_t - \int_S [\mathrm{B1m}]ds,\ B1_t = 3000$ molecules/cell.

The surface area and volume of the cell (ellipsoid with major axis 2 μm and minor axis 1 μm) were $SA = 21.5\ \mu\mathrm{m}^2$ and $V = 8.4\ \mu\mathrm{m}^3$.

The rate constants are listed below:

$k_{RL} = 2 \times 10^6\ \mathrm{M}^{-1}\ \mathrm{s}^{-1}$; $k_{RLm} = 1 \times 10^{-2}\ \mathrm{s}^{-1}$; $k_{Rs} = 4$ (molecules) s^{-1}/SA; $k_{Rd0} = 4 \times 10^{-4}\ \mathrm{s}^{-1}$; $k_{Rd1} = 4 \times 10^{-4}\ \mathrm{s}^{-1}$; $k_{G1} = 1$ (molecules)$^{-1} s^{-1} \times SA$;
$k_{Ga} = 1 \times 10^{-5}$ (molecules)$^{-1}$ $\mathrm{s}^{-1} \times SA$; $k_{Gd} = 0.1\ \mathrm{s}^{-1}$; $k_{24cm0} = 0.04\ \mathrm{s}^{-1} \times V/SA$; $k_{24cm1} = 3.3 \times 10^{-3}$ or 3.3×10^{-2} (molecules)$^{-1}$ $\mathrm{s}^{-1} \times V$; $k_{24mc} = 1\ \mathrm{s}^{-1}$; $k_{24d} = SA/3000\ \mathrm{s}^{-1}$;
$k_{42a} = 1 \times 10^{-5}$ (molecules)$^{-1}$ $\mathrm{s}^{-1} \times SA$; $k_{42d} = 0.02\ \mathrm{s}^{-1}$;
$k_{B1cm} = 1 \times 10^{-5}$ (molecules)$^{-1}$ $\mathrm{s}^{-1} \times V$; $k_{B1mc} = 0.01\ \mathrm{s}^{-1}$; $k_{Cla4a} = 0.006\ \mathrm{s}^{-1}$; $k_{Cla4d} = 0.01\ \mathrm{s}^{-1}$.

$D_s = 0,\ 0.001,\ 0.01,$ or $0.1\ \mu\mathrm{m}^2/\mathrm{s}$.
